# Films for Wound Healing Fabricated Using a Solvent Casting Technique

**DOI:** 10.3390/pharmaceutics15071914

**Published:** 2023-07-09

**Authors:** Fabiola V. Borbolla-Jiménez, Sheila I. Peña-Corona, Sonia J. Farah, María Teresa Jiménez-Valdés, Emiliano Pineda-Pérez, Alejandra Romero-Montero, María Luisa Del Prado-Audelo, Sergio Alberto Bernal-Chávez, Jonathan J. Magaña, Gerardo Leyva-Gómez

**Affiliations:** 1 Laboratorio de Medicina Genómica, Departamento de Genómica, Instituto Nacional de Rehabilitación Luis Guillermo Ibarra Ibarra, Ciudad de México 14389, Mexico; fvbj@hotmail.com (F.V.B.-J.); soniafarah1d@gmail.com (S.J.F.); teresajimenez00@gmail.com (M.T.J.-V.); emilianopineda_perez@hotmail.com (E.P.-P.); 2 Tecnologico de Monterrey, Campus Ciudad de México, Ciudad de México 14380, Mexico; luisa.delprado@tec.mx; 3 Departamento de Farmacia, Facultad de Química, Universidad Nacional Autónoma de México, Ciudad de México 04510, Mexico; sheilairaispc@gmail.com (S.I.P.-C.); alejandra.romero.montero@outlook.com (A.R.-M.); 4 Departamento de Ciencias Químico-Biológicas, Universidad de las Américas Puebla, Ex-Hda. de Sta. Catarina Mártir, Cholula 72820, Puebla, Mexico; q901108@hotmail.com

**Keywords:** skin, wound, wound healing, wound dressings, polymers, films, solvent casting

## Abstract

Wound healing is a complex process that involves restoring the structure of damaged tissues through four phases: hemostasis, inflammation, proliferation, and remodeling. Wound dressings are the most common treatment used to cover wounds, reduce infection risk and the loss of physiological fluids, and enhance wound healing. Despite there being several types of wound dressings based on different materials and fabricated through various techniques, polymeric films have been widely employed due to their biocompatibility and low immunogenicity. Furthermore, they are non-invasive, easy to apply, allow gas exchange, and can be transparent. Among different methods for designing polymeric films, solvent casting represents a reliable, preferable, and highly used technique due to its easygoing and relatively low-cost procedure compared to sophisticated methods such as spin coating, microfluidic spinning, or 3D printing. Therefore, this review focuses on the polymeric dressings obtained using this technique, emphasizing the critical manufacturing factors related to pharmaceuticals, specifically discussing the formulation variables necessary to create wound dressings that demonstrate effective performance.

## 1. Introduction

A wound is a disruption of the continuity of body tissue caused by physical or chemical damage. Usually, a wound is disinfected using a typical medical procedure, and antibiotic treatment is initiated to start the healing process [1]. However, a possible lack of response to antibiotics or an uncontrolled inflammatory phase can trigger the generation of chronic or infected wounds, which represents a clinical challenge [2]. In this sense, developing minimally-invasive smart dressings that integrate drug release with different therapeutic targets (such as antibiotics, anti-inflammatory agents, and analgesics) represents a desirable alternative [3,4].

Based on understanding the physiological process of wound healing, the ideal dressing should be biocompatible, acting as a physical barrier against microorganisms while allowing gas permeation to keep the wound hydrated and remove excess exudate [5,6]. Additionally, desirable properties include good mechanical strength and flexibility. Non-toxicity, biocompatibility, and biodegradability are also important criteria for materials used in dressings [7]. Hydrogels, polymer films, foams, gauzes, and hydrocolloids are among the most extensively studied dressings, depending on the wound type and therapeutic needs [8,9].

Films serve as convenient physical barriers to bacteria, maintain gas permeability, and enable in situ drug release. Furthermore, their flexibility can be tailored to accommodate individual morphology [10]. Consequently, research focuses on utilizing film dressings as drug carriers to control infections and inflammatory processes [11]. By providing a moist environment, removing wound exudates, and accelerating cellular and tissue regeneration, dressings can maintain optimal conditions for wound repair [12]. However, it is important to acknowledge the limitations of these products. For example, they encounter difficulties in simultaneous application to both external and internal wounds. Additionally, their application can exert external pressure, leading to secondary injuries [13].

This study aims to analyze the solvent casting procedure, the most widely used method for film production. Solvent casting is preferred over other methods, such as salt leaching, spin coating, microfluidic spinning, and 3D printing, due to its cost-effectiveness, simplicity, practicality, and ability to generate robust films with appropriate mechanical properties and homogeneity. However, the properties of materials obtained through solvent casting can vary significantly between production batches, influenced by environmental conditions, which can also slow the production process. Moreover, maintaining sterility throughout all manufacturing steps poses a challenge and can result in batch contamination.

Thus, this study comprehensively reviews the objectives of developing polymer films and highlights the key considerations to ensure optimal final properties during the solvent casting process. Despite the method’s considerable potential for industrial scaling and clinical application, there is a noticeable lack of standardized commercial products [7]. Therefore, this review identifies areas for improvement and underscores the main advantages of utilizing this type of dressing in wound treatment.

## 2. Wound Healing

**Figure 1:**
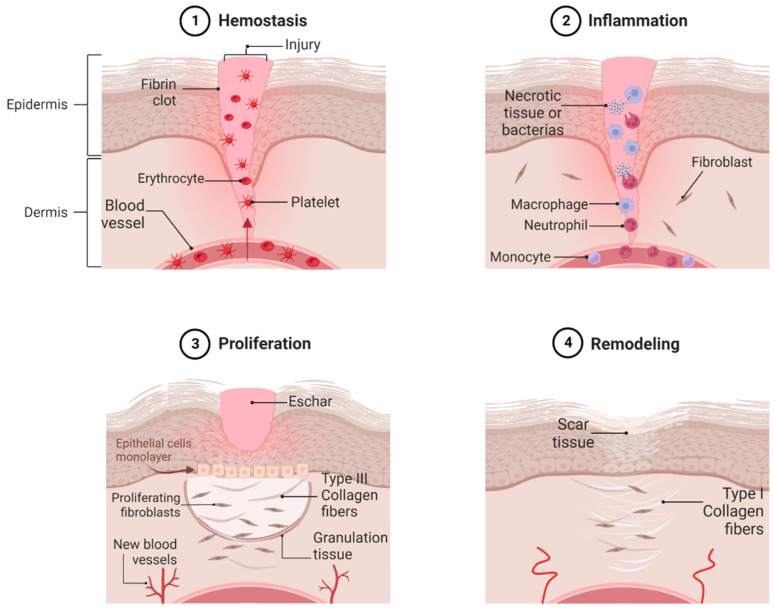
Four significant phases represent the wound healing process: (1) hemostasis, the formation of a platelet seal that prevents blood loss and a fibrin clot; (2) inflammation, where neutrophils and macrophages remove debris and prevent infection; (3) proliferation, where blood vessels reform through angiogenesis, and fibroblasts replace the fibrin clot with granulation tissue; (4) remodeling, where the matrix is remodeled replacing type III collagen with type I, maturing to a scar.

The wound healing process is a natural physiological reaction to tissue injury that consists of four highly integrated phases: hemostasis, inflammation, proliferation, and remodeling (Figure 1). These phases must occur in a specific sequence, at a specific time, and for a particular duration, in order that the physiologically involved functions fulfill their expected role [14]. Otherwise, this leads to improper or impaired tissue repair [15].

### 2.1. Hemostasis

The hemostasis phase begins minutes to hours after an injury through a cascade of serine protease activation, resulting in platelet activation. This activation also facilitates the release of growth factors, such as PDGF (platelet-derived growth factor), VEGF (vascular endothelial growth factor), and TGF-α (transforming growth factor α), as well as immune mediators, contributing to the transition into the inflammatory phase [16]. PDGF and TGF-β (transforming growth factor β) recruit neutrophils and monocytes to initiate the inflammatory response [17]. Furthermore, this leads to the formation of a fibrin clot, referred to as an eschar, which acts as a plug for the wound, preventing blood loss, providing a scaffold for incoming immune cells, serving as a reservoir for cytokines and growth factors during the early stages of repair, and offering protection against bacterial invasion [15,16,18].

The extrinsic clotting cascade is initiated upon tissue damage and blood leakage, releasing molecules such as serotonin that induce localized vasoconstriction [19]. Subsequently, platelets aggregate and become activated upon contact with subendothelial collagen, forming a hemostatic plug. This plug mitigates hemorrhage and serves as a provisional matrix for cell migration by releasing scaffold proteins such as fibronectin, vitronectin, and thrombospondins, facilitating the migration of keratinocytes, immune cells, and fibroblasts [16,20]. Once a fibrin clot is formed, the coagulation process is switched off to prevent excessive thrombosis [17].

### 2.2. Inflammation

The inflammatory phase, which overlaps with hemostasis, occurs during the first 72 h [16]. During this phase, both humoral and cellular inflammatory responses are activated to establish an immune barrier against invading microorganisms [21]. Neutrophils and monocytes infiltrate the wound bed to prevent further damage and eliminate pathogenic organisms and foreign debris [16,22].

The inflammation phase can be divided into early and late inflammatory phases. In the early phase, the complement cascade is activated, leading to the infiltration of neutrophils into the wound. Their primary role is to phagocytose bacteria, foreign particles, and damaged tissue [21,23]. Various chemoattractive agents attract neutrophils to the wound site, including TGF-β, complement components such as C3a and C5a, and formyl-methionyl peptides produced by bacteria and platelet products [16,19]. During their activity, neutrophils release proteolytic enzymes, oxygen-derived free radical species, and inflammatory mediators such as TNF-α and interleukin (IL)-1 [17,21]. Once their role is fulfilled, neutrophils undergo apoptosis and are cleared from the wound typically within 2–3 days, making way for the influx of monocytes [18].

Monocytes, stimulated by cytokines, chemokines, growth factors, and soluble fragments of the extracellular matrix (ECM), differentiate into activated macrophages. These macrophages patrol the wound area, ingesting and killing bacteria and removing devitalized tissue through the action of secreted matrix metalloproteinase and elastase [15]. Additionally, macrophages play a crucial role in transitioning to the proliferative phase by releasing various growth factors and cytokines, including PDGF, TGF-α, TGF-β, insulin-like growth factor-1 (IGF-1), fibroblast growth factor (FGF), tumor necrosis factor-α (TNF-α), IL-1, and IL-6. These soluble mediators promote cell proliferation and the synthesis of ECM molecules, and activate fibroblasts for subsequent phases [19,24]. A decrease in macrophage presence within the wound indicates the inflammatory phase’s conclusion and the proliferative phase’s initiation [15].

### 2.3. Proliferation

The proliferative phase of wound healing involves several interconnected processes that restore tissue structure and function. It begins on the third day after the injury and lasts approximately two weeks [21]. One of the critical aspects of this phase is the replacement of the provisional fibrin matrix with a new matrix composed of collagen fibers, fibronectins, and proteoglycans, which are synthesized by fibroblasts.

During this phase, angiogenesis, the generation of granulation tissue, collagen deposition, re-epithelialization, and wound contraction occur [16,25]. Angiogenesis is stimulated by local conditions such as low oxygen tension, low pH, and high lactate levels. It involves the migration and proliferation of endothelial cells to form new blood vessels, which is crucial for tissue viability [17]. Macrophages are vital in promoting angiogenesis by producing VEGF [16].

Granulation tissue formation occurs concurrently with angiogenesis and primarily comprises type III collagen, fibroblasts, and new vessels. Fibroblasts are the main cells involved in granulation tissue formation. Their proliferation, and synthesis of extracellular matrix components, contributes to tissue restoration [21]. Additionally, the interaction between fibroblasts and keratinocytes plays a significant role in re-epithelialization. Factors like EGF, KGF, and TGF-α, produced by platelets, keratinocytes, and anti-inflammatory macrophages, promote keratinocyte migration and proliferation [25,26,27]. The process is further facilitated by the production of fibronectin, tenascin C, and laminin 332 by keratinocytes [16].

As re-epithelialization progresses, the stratified layers of the epidermis are re-established, and the maturation of the epidermis begins to restore its barrier function. TGF-*β* can accelerate this maturation process [17]. The proliferation and differentiation of keratinocytes are essential for the reestablishment of a functional epidermis [25,27].

### 2.4. Remodeling

The remodeling phase, which occurs several weeks after the initial wound, marks the transition from granulation tissue to scar formation. This phase may last up to 1–2 years [21]. During this phase, angiogenesis slows down, and type III collagen in the granulation tissue is replaced by stronger type I collagen [16].

Myofibroblasts play a crucial role in driving the remodeling phase. They originate from fibroblasts and develop in response to mechanical tension and TGF-*β* signaling. Myofibroblasts are responsible for wound contraction, and their expression of smooth muscle actin (SMA) generates the contractile force exhibited by these cells [28,29]. It is worth noting that myofibroblasts are considered terminally differentiated and undergo apoptosis once the remodeling process is complete [16].

During the remodeling phase, the granulation tissue matures into a scar, and the tensile strength of the tissue increases. This maturation is characterized by a reduction in the number of capillaries, an aggregation into larger vessels, and a decrease in the number of glycosaminoglycans. The cell density and metabolic activity in the granulation tissue also increase during this maturation process. The collagen type and organization changes further enhance the tissue’s tensile strength [17].

However, the tensile strength of the healed tissue never fully reaches its original strength. A newly epithelialized wound typically has approximately 25% of the tensile strength of normal tissue, and it may take several months for the tensile strength to increase to a maximum of 80% of normal tissue [30]. The enhancement of tissue tensile strength primarily occurs through reorganizing collagen fibers, initially deposited randomly during the granulation phase. After, the enzyme lysyl oxidase, secreted by fibroblasts into the extracellular matrix (ECM), facilitates the increased covalent cross-linking of collagen molecules [17].

## 3. Polymeric Films for Wound Healing

Polymeric films (PFs) as dressings for wound healing were first vastly introduced during the Second World War as a response to the demand for advancements in medicine [31]. Bloom et al. [17] were the first to document semipermeable films in 1945 using cellophane to treat burnt prisoners of war in Italy; they reported “gratifying results” with complete healing nine days after dressing application [32].

Nowadays, PFs are commonly utilized in the medical field for healing as physical barriers for wounds which help to prevent inflammation, control the environment of the wound, and accelerate healing [33]. PFs have gained popularity due to their non-invasive nature, ease of application, biocompatibility, and the potential inclusion of antimicrobial treatments. Moreover, PFs offer flexibility, adherence, gas exchange capabilities, and transparency [34]. The material’s flexibility enables the film to conform to complex shapes while facilitating gas exchange, which has been proven to promote healing [34]. Additionally, its transparency allows for the close monitoring of the wounded area without removing the film, thereby reducing trauma during dressing changes, minimizing exposure to bacteria, and lowering the risk of infection by up to one week [34,35].

PFs are recommended for treating partial-thickness wounds, minor burns, lacerations, and certain low-exudate ulcers such as ischemic ulcers, diabetic ulcers, venous ulcers, and pressure ulcers [34]. However, it is important to note that due to the occlusive nature of the material, films should not be used on wounds that require significant fluid absorption, as excess exudate can potentially lead to peri-wound maceration. Nevertheless, maintaining a certain level of moisture can be beneficial for the healing process by promoting keratinocyte migration to the affected area [34,36].

The performance of PFs depends on the chemical composition and the type of wound. Polymers used to formulate PFs can be divided into natural, synthetic, and blended. Therefore, the PFs will possess different qualities depending on the type of polymers employed to form the film dressing.

### 3.1. Natural Polymeric Films

Natural polymers, also called biopolymers, offer advantages such as biocompatibility, biodegradability—at present a sought-after quality—healing properties, inertness, and adhesiveness [37]. For this reason, generally, natural polymers are preferred over synthetic ones [37]. However, they are prone to microorganism contamination and lack quality mechanical properties to form an optimal polymeric film.

**Figure 2:**
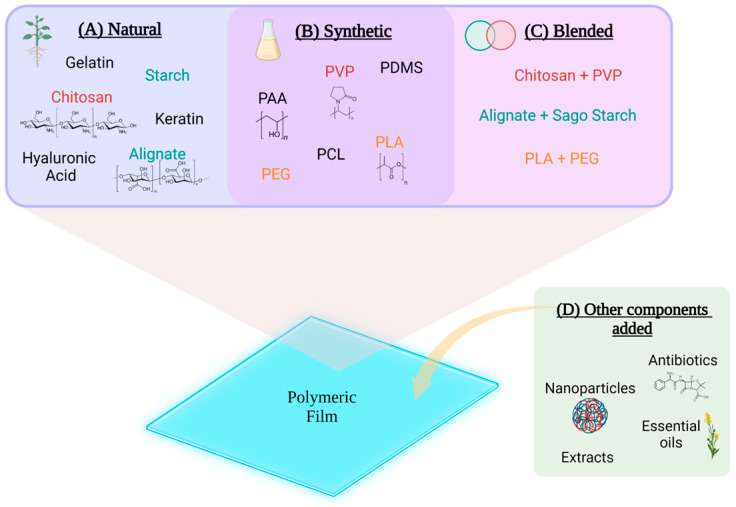
Composition of polymeric films. Examples of some polymers that may be used to form polymeric films. (**A**) Natural polymers; (**B**) synthetic polymers; (**C**) brief examples of blended polymers; (**D**) other components that can be added to enhance PFs’ characteristics.

Some examples of natural polymers used for elaborating PFs are chitosan, hyaluronic acid, starch, silk fibroin, sericin, keratin, sodium alginate, gelatin, collagen, zein, and konjac glucomannan, among others (Table 1) [36]. Chitosan is one of the most exploited and abundant natural polymers in wound dressings, with antimicrobial and film-forming properties [38,39]. Moreover, due to their innocuousness, polymers like chitosan, cellulose, gellan gum, alginates, and starches can be used for oral cavity films [40] (Figure 2).

### 3.2. Synthetic Polymeric Films

Unlike their natural counterparts, synthetic polymers can provide better mechanical properties such as strength, flexibility, structure, and a higher degree of polymerization. However, these polymers lack biocompatibility, which may cause immunological reactions in the body, rejecting the film, may have low adherence to the wound site, and have less absorption and permeability [37] (Table 1).

Synthetic PFs can be either passive or interactive. Passive films are non-occlusive and function as a cover for the wound. On the other hand, interactive films can be semi-occlusive or occlusive and provide a barrier against bacteria from the wounded area. Semi-occlusive films can allow gas exchange, which provides better healing conditions, but can still be permeable to bacteria, for example, polyurethane films. It is considered that occlusivity and impermeability to bacteria are desirable in a wound dressing, for it mimics the skin. However, due to their lack of absorbance, semi-occlusive and occlusive films have the drawback that exudates may seep through the material [38,44]. Some synthetic polymers commonly used in PFs are polyvinyl alcohol (PVA), polyacrylic acid (PAA), poly-ε-caprolactone (PCL), polyethylene glycol (PEG), polyvinylpyrrolidone (PVP), polylactic acid (PLA), and polydimethylsiloxane (PDMS), among others (Figure 2) [36].

### 3.3. Blended Polymeric Films

Blended PFs combine multiple polymers and can be synthetic and natural. By blending different polymers, a film with the advantages of both polymers can be attained, resulting in a PF with more desirable physicochemical properties and better mechanical characteristics [33,45,46] (Table 1). Achieving compatibility is crucial when blending polymers because, otherwise, the resulting mixture may exhibit deteriorated properties [40]. Combining different polymers allows for new opportunities to develop better biomaterials for specific wound healing [33]. The number of polymer combinations can be factorial, with infinite possibilities according to the application requirements. Even new derivatizations of synthetic and natural polymers increase the range of options, combined with the plasticizing agent, which is almost always essential in forming the film.

Although the polymers used in PFs have properties for wound healing, such as antimicrobial and antioxidant properties, they can be limited [45]. As a result, other components are added to enhance healing, like natural compounds (curcumin, extracts, polyphenols, and essential oils), antibiotics, and nanoparticles for controlled release, among others (Figure 2) [36]. For example, PFs can be used as a dosage form for nanoparticles to create drug delivery systems that control the drug release at the wound site and provide a better control of drug concentration and delivery for a specific time [34].

## 4. The Solvent Casting Method

The production of PFs requires casting or preparation methods that directly impact the film properties [47]. These techniques are unique and tailored to specific polymers and application areas [48]. The desired properties of PFs depend on their intended use, which can range from food packaging to tissue engineering and medical applications [49]. In this review, comprehensive research was conducted, focusing on the preparation methods of PFs for wound healing.

As mentioned, the most utilized preparation methods include salt leaching, spin coating, microfluidic spinning, 3D printing, and solvent casting. Each method comes with its own set of advantages and disadvantages. The salt leaching method relies on the insolubility of inorganic salts in organic solvents that dissolve biodegradable polymers. It involves preparing a polymer solution, adding a pore former, pouring the mixture onto Petri dishes, and washing it with deionized water to leach out the salt crystals. The resulting PF is porous [36]. The main advantage of this method is its high porosity, while its main disadvantage is the limitation of using water-soluble materials, as they can leach out along with the salt [50].

The spin coating method involves depositing uniform polymer layers by dispersing them on a rotating plate, resulting in thin layers of 1–10 µm thickness. The rotation facilitates solvent evaporation, and the speed can be adjusted to control the final thickness [51]. The main advantage of spin coating is its versatility in modifying the thickness of the final PF. However, its low material efficiency is its drawback, as it utilizes only 2–5% of the dispensed material [52].

Microfluidic spinning operates on the principle of microscale fluid dynamics, where core and sheath flows create a coaxial flow within a specially designed microchannel. The polymer dispersion is solidified through UV light, ionic or chemical crosslinking, and solvent exchange [36]. The notable advantage of this method is its ability to form 3D fibrous structures by manipulating individual fibers. However, it is associated with higher costs and energy consumption [53].

The process of 3D printing involves the extrusion of suitable polymer solutions to create biocompatible PFs. It offers the advantage of producing flexible matrices with predetermined pore sizes in a reproducible manner. However, this method is relatively expensive and requires specialized equipment [36].

**Figure 3:**
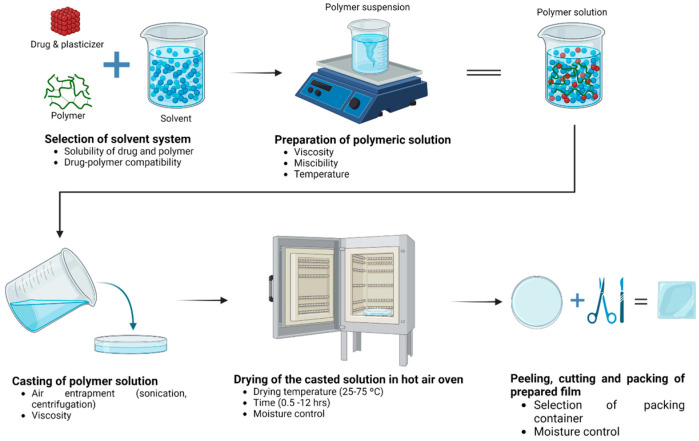
Solvent casting method for film production with quality control parameters. The process begins with selecting the polymer and solvent to prepare the polymeric solution, which is then poured into a mold. The mold is left to dry in an oven, and finally, the film is peeled, cut, and packaged.

Among all the casting techniques available, the solvent casting method represents a reliable, preferable, and highly-used method due to its easygoing and relatively low-cost procedure [54]. The solvent casting method is a process that involves the solubilization of a polymer and plasticizer, the spreading of the solution on a substrate, and solvent removal, which causes the molecular orientation of the polymer chains and the intercalation of plasticizer molecules, resulting in film formation [55]. In this method, the polymer(s) and plasticizer(s) are dissolved in a volatile solvent, like ethanol, acetone, water, or a combination of solvents. If added, a drug can be suspended or dissolved in the solution, cast into a mold, and left to dry (Figure 3).

### 4.1. Advantages

The solvent casting methodology has multiple advantages, such as improved physicochemical properties, easiness, low-cost processing, and adequate thickness uniformity. This approach is also employed in producing films that incorporate heat-sensitive active pharmaceutical ingredients (APIs), as the necessary temperatures for eliminating the solvent are comparatively low [56]. It also enables the tunability of the mechanical and optical properties of the film through the variation of processing parameters, such as solvent casting time and temperature, hence allowing the production of films with high optical clarity and porosity (Figure 3) [57]. Therefore, the solvent casting method is a robust process that is easy to scale up industrially.

Regarding wound healing, some advantages that the solvent casting method provides are desirable, such as transparency, impermeability, porosity, cost-effectiveness, and flexibility. Transparency allows for a better assessment of the wound, no need for frequent wound dressing removal, and lower infection rate and trauma during dressing changes. Porosity or moisture vapor and oxygen permeability inhibit the growth of anaerobic bacteria transmitting water vapor from beneath the dressing to the external environment. Impermeability to microorganisms and moisture inhibits the growth of bacteria and serves as a physical barrier to water and bacteria. Also, the wound dressing may help to create a moist wound environment and induce autolytic debridement [33,36,58,59,60]. Moreover, the solvent casting method yields better results for wound-healing dressings than the previously mentioned methods. For example, solvent casting nanofibers were more appropriate than the bilayered carrier on a 3D-printed layer [61].

Nonetheless, some disadvantages should be considered. The most noticeable is the excessive use of solvent for the process, making it harder and relatively expensive to produce these dressings on an industrial scale. Other demerits are the long drying time and the lack of film thickness control [48].

### 4.2. The Importance of the Polymer

Commonly, polymers are seen as excipients, but they have become an essential component for the formulation of wound healing films. Polymers constitute the backbone of film formulations; consequently, it is crucial to understand the polymers’ chemical, physicochemical, and rheological properties. Furthermore, selecting an appropriate polymer during film development is critical to ensure therapeutic success [54].

Polymers in PFs are impermeable to bacteria and liquid but are permeable to moisture vapor and air. Also, they may trap exudates, providing a moist environment for wounds [56]. Biopolymers can also provide anti-inflammatory, cell proliferative, and angiogenic effects, leading to a favorable niche in the healing process and a drug release platform [56,62]. Other polymers can counteract the presence of opportunistic microorganisms. However, since most polymers tend to produce brittle films, incorporating a plasticizing agent is almost always necessary. Some examples of biopolymers used for wound healing are shown in Table 2.

### 4.3. The Influence of the Plasticizing Agent

PFs, especially those of natural origin, tend to degrade rapidly with the environment and have physical limitations like low mechanical resistance and poor flexibility. As a result, additives are included in the film composition (Table 2).

Plasticizers are non-volatile compounds commonly used as additives, primarily aimed at enhancing the flexibility and processability of polymers by reducing the glass transition temperature (Tg), which is the temperature at which polymers transition from a rigid to a more flexible state. Plasticizers weaken intermolecular forces between polymer chains, increasing free space and chain mobility [68]. Consequently, they decrease tension deformation, hardness, viscosity, and the electrostatic charge of polymers. Plasticizers also impact properties like crystallinity, optical clarity, electrical conductivity, and resistance to biological degradation [68]. These compounds typically have a low molecular weight (around 200 to 400 g/mol), can be hydrophilic or lipophilic, and exist in solid or liquid form. A combination of plasticizers can be used, and their effect on mechanical properties depends on their type and concentration.

Plasticizers are generally used in concentrations ranging from 5 to 30% (*w*/*w*), although an optimal range can be determined through experimental design. It is important to note that using a plasticizer in low concentrations can lead to the opposite effect, increasing the polymer’s rigidity, known as antiplasticization or the low plasticizer concentration effect [69].

Common hydrophilic plasticizers include glycerin, polyethylene glycol, propylene glycol, and sorbitol (Table 2). Lipophilic plasticizers commonly used include acetyl tributyl citrate, acetyl triethyl citrate, dibutyl sebacate, diethyl phthalate, triacetin, tributyl citrate, and triethyl citrate. Some drugs may have plasticizing properties; even water can be a plasticizer. However, PFs with a low percentage of water, even with an adequate plasticizer concentration, may not exhibit satisfactory mechanical properties and drug release behavior [70].

The presence of a plasticizer generally increases the flexibility of PFs while decreasing their elastic modulus and tensile strength. Nevertheless, specific interactions may enhance the flexibility, elastic modulus, and tensile strength. When selecting a plasticizer to produce PFs intended for wound healing, factors like plasticizer toxicity, compatibility with the polymer and the drug or bioactive component, influence on drug release, and impact on the film’s mechanical properties should be considered. Other important considerations include the ease of handling and cost-effectiveness [71].

### 4.4. Blend of Polymers

Considering that the importance of the polymer selection lies in the intended application, it is relevant to mention that combining two or more polymers to obtain blends is a simple and cost-efficient strategy for developing PFs with tuned properties [70]. The essential advantages of polymer blending are the new or improved properties for specific needs, material cost reduction with little or no loss of properties, material processability improvement, and avoiding the polymerization step [72]. It is possible to combine natural, synthetic, or a combination of natural and synthetic polymers; in most cases, the feasibility is determined by the solubility affinity.

Polymer blends are called “compatible” or “incompatible”, depending on their resulting properties. Compatible blends exhibit delicate phase morphology resulting in good physical properties. Usually, the chances of obtaining synergistic properties are high in a consistent mix. On the other hand, incompatible blends are fully immiscible and have poor mechanical properties [72].

One of the most studied blends of polymers is alginate with chitosan. When chitosan and alginate, which have an opposite charge, are mixed in an aqueous phase, they spontaneously combine owing to a strong electrostatic attraction and form a polyelectrolyte complex (PEC), conferring better mechanical stability and resistance to pH variations than either chitosan or alginate separately [71].

### 4.5. Polymer–Polymer, Drug–Polymer Incompatibility Phenomena

The previously mentioned blends and their improved combined effects occur due to polymer-polymer interactions. The emergence of blend-based films has improved physical, transport, and mechanical properties compared to single-component films [73]. In a polymer blend, the interactions between the different monomers are predominantly hydrogen bonding, Van der Waals interactions, or dipole–dipole interactions [72]. Polymer–polymer incompatibility appears when there is low miscibility in a common solvent during the dissolution process, or no miscibility of one polymer in another during the casting process. Then, the type of polymer, solubility, proportion, functional groups present in the monomer, and degree of crystallinity will be decisive.

A similar phenomenon occurs in drug–polymer incompatibility processes. To our knowledge, most incompatibility phenomena occur by immiscibility in solution; this aspect can be prevented in most cases. However, the proportion of the components will be decisive, considering that in most formulations, there will be at least one polymer, one plasticizer, one drug, and one solvent; then, there may be competition regarding solubility. Low-potency drugs with high required doses can bring the dissolution system and PF molding process to the saturation limit. Drugs can be crystallized in the polymer when not miscible, reducing apparent solubility and decreasing the dissolution rate. The degree of miscibility between the drug and polymer is vital for solubility enhancement and forming a physically stable amorphous system [74]. Another critical factor when delivering APIs through films is that some drugs have poor solubility. Therefore, the use of an amorphous solid dispersion (ASD) (being the biopolymer film itself) enhances the aqueous dissolution performance of a drug without the need for chemical modification [75].

### 4.6. Growth of Microorganisms

Although part of the purpose of the PFs is to prevent wound contamination, it should be noted that the growth of microorganisms in a polymer solution or during the storage of a PF can be attributed to non-sterile conditions. Diverse strategies have been used to prevent microbial proliferation on the wound healing film. The development of films predominantly aims to prevent microbial adhesion and biofilm formation. Such materials either repel microbes (antifouling) or kill bacteria (antimicrobial).

Antifouling PFs avoid the use of drugs, but their efficacy depends on the bacterial species. As bacterial surfaces are mainly hydrophobic and negatively charged, the antifouling polymer must be (1) hydrophilic, (2) negatively charged, or (3) with low free surface energy [76]. Among hydrophilic polymers, polyethylene glycol (PEG) has proven antifouling properties related to hydration and steric effects [77].

Antimicrobial PF can be achieved by introducing the polymer backbone side-chain functionalities or by impregnating antimicrobial agents. Cationic polymers (CPs), like chitosan, carry positive charges in the primary or side chain. These water-soluble polymers establish solid electrostatic interactions with the bacterial cell membrane, causing cell wall and/or membrane disruption, the leakage of intracellular material, and cell death [78]. Some examples of novel antimicrobial molecules on wound healing films are curcumin and epigallocatechin gallate (EGCG) [66,79]. Both molecules are known to have potent antioxidant and free radical scavenging activities. They also inhibit the growth of bacteria in various ways, including by the disruption of cell membranes through interaction with surface proteins, decomposition of essential metabolites, inhibition of relevant enzymes, induction of ROS stress, change in cell wall structure, and detachment of the cytoplasm [80].

During the fabrication process, the drawback of microorganism growth can be avoided by ensuring sterile conditions, intermediate sterilization, or terminal sterilization through non-ionizing or ionizing radiation methods. Subsequently, the PF for application in wound healing must be presented as a product in a hermetically sealed envelope to guarantee stability and sterility during its shelf life.

## 5. Fabrication

### 5.1. The Solvent Process

The solvent casting technique involves a first stage where the polymer, plasticizers, and drug are dissolved in a suitable solvent to be later molded into an appropriate geometric shape. The principles governing any chemical mixing process can be applied, being evident that the polymer must be completely soluble in the selected volatile solvent or water. The solution must contain minimal solids and an equally reduced viscosity [81,82,83]. Homogeneous solutions leading to obtaining materials with desirable mechanical properties are frequently obtained using co-solvent systems, dissolutions under pressure, specific polymers with suitable molecular weight distributions, or copolymers [84,85]. Polystyrene and poly (isobutyl methacrylate) coatings cast from toluene have shown that residual internal stress is independent of initial solution concentration [86]. Shrimp chitosan films were prepared using organic acids (acetic, lactic, maleic, tartaric, and citric acids) to solubilize chitosan, and, according to Eulálio et al. (2020) [57], depending on the organic acid used, the physicochemical properties, degradation, and cytotoxicity of the films are modified [87].

**Figure 4:**
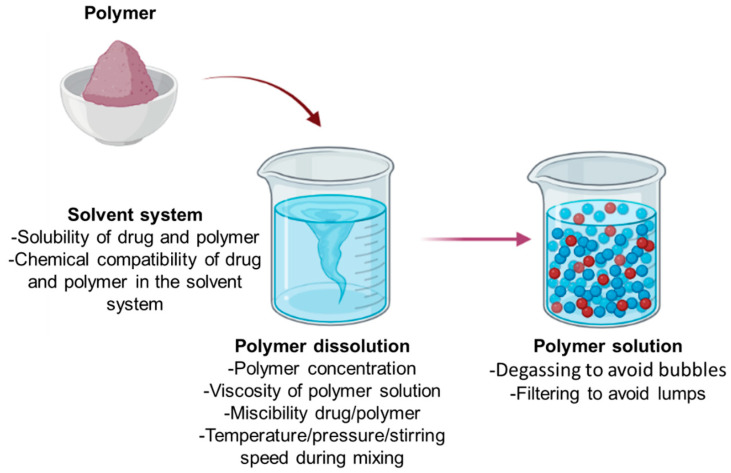
The solvent process.

Regarding the viscosity, typical values range from 1500 to 80,000 kPa in the dissolution process, while the concentration of solids ranges from 5 to 40% (*w*/*w*), and the temperature varies from room temperature to below the boiling point of the solvent (Figure 4) [88,89,90]. For example, chitosan/hyaluronic acid films, prepared with an increased concentration of the polymers (35%), formed aggregates; water retention within the film was consequently favored, limiting permeation, and reducing the elasticity and flexibility of the films [91].

For polymers that are difficult to dissolve (i.e., PVC), it is common to use instruments that allow the increase of pressure and temperature. The mixture is degassed by boiling after complete dissolution or placing it under a vacuum to avoid bubbles during film formation (Figure 4) [92]. After the dissolution process, it is usual to filter the solution to prevent the discharge of polymer clusters [93]. Using water as a solvent is common to obtain food-grade materials or for medical applications [94,95,96].

Another strategy is the addition of excipients that favor appropriate crystalline states or prevent the agglomeration of polymer chains [97]; for example, Prest et al. (1979) [65] identified that the orientation of the plasticizer functional groups influences the direction of the polymer chains and that this directly influences the optical properties of the resulting films [98]. Also, Ghosal et al. (2018) [66] demonstrated that membranes cast with the same solvent and with semi-crystalline domains were brittle and inflexible in contrast to amorphous membranes, evidencing that the crystalline state of the polymer in solution is determinant in the resulting properties [99].

Additional essential factors during the dissolution process are the type of stirrer, stirring speed, and temperature needed to avoid gel particle formation, uncontrolled solvent evaporation, early film formation, and polymer degradation [54,100,101]. For example, Boateng et al. (2009) [54] employed vortex agitation for a faster dissolution of carboxymethyl cellulose, avoiding lumping, reducing viscosity, and quickly releasing trapped air bubbles, resulting in clear and transparent solutions that are easily poured into molds [84]. In addition, despite the decrease in film ductility due to the loading of the drug as a powder, the produced film exhibited adequate flexibility and resistance to elongation, along with rapid dissolution [102].

Undoubtedly, the type of agitation is crucial in order to minimize the formation of bubbles so as not to alter the physical appearance and mechanical performance. Depending on the viscosity of the solution, ultrasound can help to reduce the presence of bubbles in small-scale batches, and the centrifugation at low speeds (approx. 1000–5000 rpm) and times (approx. 3–5 min) favors the displacement of residual air. In processes where the operation time is not critical, the rest of the solution displaces the residual air due to gravity phenomena in low- and medium-viscosity systems. The rest of the solution can be at room temperature or low temperature. Finally, it is essential to consider the type of rheology generated in the formation of the solution to adapt the most convenient type of agitation.

### 5.2. The Casting Process

The solvent casting method is the manufacturing method of choice for films because of its relative simplicity and low costs [103]. However, this method could be limited for industrial scale-up because it is usually associated with large amounts of organic solvent required to dissolve the polymer’s discharge volume [46].

In the solvent casting method, all ingredients are dissolved in a suitable solvent, cast onto a release liner, and dried, cut, stripped, and packed [103]. When the solvent is constantly added to the solid polymer, the swollen surface layer grows until it reaches a nearly stable state. This increase in layer size limits the movement of large molecules from the surface into the solution [46].

Polymer pouring is related to mechanical properties. For example, Tu et al. (2014) [72] reported two bilayer matrix configurations, leaching either alone (6% *w*/*v*, silk fibroin solution mixed with NaCl) or in combination (NaCl/silk solution layered onto a silk film generated first by drying an 8% *w*/*v*, silk solution). The authors registered the functional support tissue, and the scaffolds with a silk film displayed a significantly high initial elastic modulus and ultimate tensile strength compared to the matrix alone [104].

Usually, glass, plastic, stainless steel, or Teflon plates are used as inert bases for film casting. The choice of a container depends on the specific characteristics of the film and can range from single-unit containers to multiple-unit dispensers [105].

**Figure 5:**
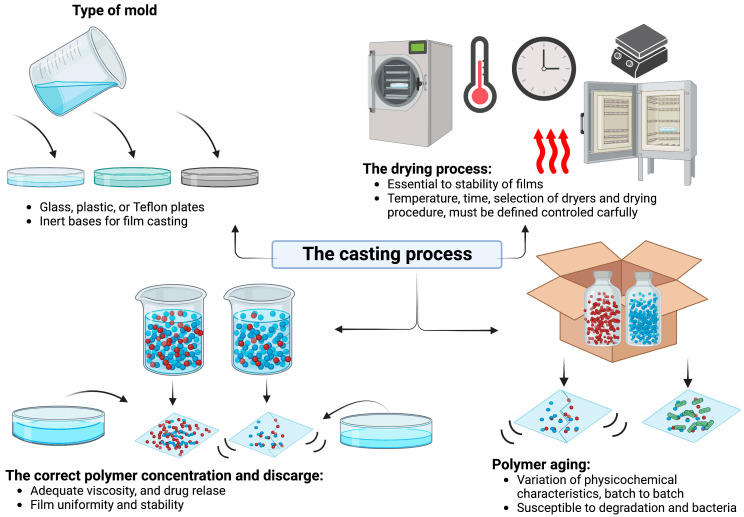
Factors implicated in the casting process. The type of mold, the drying process, the polymer age, the correct polymer concentration, and discharge are procedures related to an adequate drug release for films for wound healing through the solvent casting technique.

Also, the drying process (temperature and time) is essential to the stability of the films and must be defined and controlled carefully. Therefore, optimizing the casting and drying operation speeds has the most vital direct influence on production and the commercial scale output, limiting the product’s drying speed and final thickness [106]. High drying speeds can increase bubble formation, while a decrease in speed can even be carried out at room temperature. Therefore, the polymeric film’s moisture content and thickness are some of the main quality control parameters in the molding process (Figure 5).

A proper bow-type or horizontal-nozzle, indirect heating, heating by radiation, freeze-drying, airstream drying, or a hot-flue type dryer separated or combined in different zones to increase the accuracy of control over film formation, the film drying at the support surface, and the number of on-line dryers all improve the processing times; the greater the efficiency in the removal of hot, humid air, the greater the speed of drying [71,73,74].

Usually, the drying procedure is prolonged for small-scale production; the slow production speed depends on a slow solvent diffusion process and facilitates the growth of bacteria [103]. A high drying temperature reduces the drying time but influences the films’ stability. Drying with hot air at a high-speed causes solvents to evaporate immediately, with no uniformity in the film. An option is freeze-drying in aqueous gels or polymers. However, it is a complex and expensive procedure and can stabilize some bacteria during freeze-drying [107].

One prerequisite for the polymer solution casting process is that the polymer must be soluble in a volatile solvent or water. The type of polymer, the content, and the concentration are essential to achieve adequate viscosity. A stable solution with a reasonable minimum solid content and viscosity should be formed [46]. In this method, the problems that may occur during the manufacturing process include unsuitable casting solutions due to inappropriate viscosity and poor content uniformity [103]. The combination of polymers in a single formulation can improve mechanical properties. However, the polymer, plasticizer, and drug interactions can cause instability phenomena, cannot be described in general, and need individual investigations for each polymer or combination of polymers [103].

In drug formulations, the release of the drug is affected by the viscosity and concentration of the polymer used. The interaction between the polymer and drug is crucial in creating a stable, continuous network and cohesive film [46,108]. For example, fast-dissolving films of loratadine using low viscosity grades of HPMC as film-forming polymers show that 300 o 600 mg of HPMC E3 in the formulation released the complete drug in 6 min as compared with the use of 300, 600, or 900 mg HPMC E15, which was unable to release the drug entirely within 10 min due to its increased polymer viscosity [108]. Polymers with higher molecular weights take longer to dissolve than those with lower molecular weights. The longer chain in higher molecular weight polymers experiences a much slower relaxation rate due to greater chain entanglement, which retards the flow and formation of the advancing gel boundary [46].

Typically, viscosities change from 1500 mPa to 80,000 mPa during the dissolution process of solids [106]. The influence of polymer concentration was reported by Haq et al. (2020) [77]; in their study, the addition of 4% *w*/*w* HPMC revealed a reduction in elastic modulus compared with the addition of 6% *w*/*w* in a biocompatible topical polymeric film that has the potential to deliver the antibacterial agent directly to the skin to manage the local wound infection, and thereby wound healing [109]. The polymers undergo an aging process during casting, and potential increases in batch-to-batch variation are expected. A study conducted by Luque-Agudo et al. (2019) [78] evaluated, in vitro, the surface properties, degradation, and the bacterial response of films of poly(lactic) acid with Mg particles for 4 and 8 h and 1, 7, 14, 21, and 28 days. The authors observed that the degradation of the films allowed the appearance of a biocompatible layer composed mainly of Mg phosphates, decreasing the hydrophobicity of the film [110]. Also, aging is essential when the matrix comprises a semi-crystalline polymer susceptible to transformations [111]. The effect of the aging process on the mechanical properties and clinical performance of polymeric films for wound healing can be studied by gradually varying the temperature and humidity as a function of time.

## 6. Reproducibility, Establishing Quality Control Parameters

The solvent casting technique is appropriate for fabricating PFs for biomedical applications and is broadly used to increase drug incorporations [112,113]. This method offers relevant results regarding smoothness, high malleability, appropriate thickness, transparency, and film light transmission values [114]. Furthermore, the addition of stabilizing agents helps to improve some PFs’ properties; for instance, plasticizers improve flexibility and chain mobility while decreasing brittleness and shrinkage during usage and storage [113,115].

However, different physical parameters, such as film weight, thickness, moisture percentage, appearance, swelling capacity, water vapor permeability, and mechanical properties, mainly, can change if process controls are missing, such as quality control parameters. A change in a physical property involves a variation in functionality, with possible consequences of decreased therapeutic efficacy and the risk of compromising and delaying the wound closure process. Mainly, the drying process is one of the unit operations that can cause the most significant number of deviations. An increase in the drying rate may lead to the formation of bubbles, which will alter the appearance and may change the mechanical properties. However, controlling the speed and drying time will aid the maintenance of an adequate thickness of the PF, and, consequently, an appropriate moisture content. Likewise, the rest of the properties could remain stable.

Film thickness is a parameter that can affect the required time to absorb the polymer into the body, affecting the properties of PFs [38], and it is frequently measured using a micrometer. The acceptable thickness range may be between 0.26 and 0.841 mm [38,116]. A thin PF offers several advantages, including a faster onset of drug action, reduced dose frequency, and improved drug efficacy [116].

Swelling ability is a significant parameter since the excess of exudate should be absorbed by the PF. Although a smooth morphology is preferred, PFs with higher porosities may help to absorb more wound exudates and facilitate the distribution of nutrients and medium to the cells [117,118]. In this sense, carbohydrates such as chitosan and sodium alginate are known to be swellable in physiological fluids [38].

Water vapor permeability (WVP) is an essential property in PFs because it helps to control the water loss from the wound and to maintain a moist environment during the wound-repairing process. [115]. The water vapor transmission rate (WVTR) is crucial, providing a humid environment and gas exchange for wound healing. Wound dehydration may occur due to an excessive WVTR, whereas bacterial infection and maceration may result from low WVTR [119,120]. The WVTR for normal skin is 204 g/m^2^/24 h. However, for injured skin, it may increase from 279 g/m^2^/24 h (in first-degree burns) to 5138 g/m^2^/24 h (in granulating wounds) [121].

The mechanical properties of PFs are generally related to the film network microstructure and intermolecular forces [115]. They are crucial for their clinical performance to protect the wound and avoid tearing up while withstanding stresses during the application, handling, or break-in storage [119]. Therefore, PFs should combine relatively high strength with ductility and flexibility to follow skin movements (Table 3) [116]. However, a rapid decrease in these properties is expected to occur during application due to hydrolysis and enzymatic breakdown [119]. The mechanical properties of the PFs are usually characterized by tensile strength (TS), elongation at break (EB or strain), and Young’s modulus. The TS is the maximum stress that a film can withstand being stretched before necking or cracking [38]. EB is a measure of the film’s stretchability before breakage [115], and Young’s modulus (YM) is defined as the slope of the stress–strain curve in the elastic region [119] (Table 3). The recommended values of TS and strain for skin comprise 2.5–16 MPa and 70%, respectively [121].

The solvent used for PFs elaboration may influence its physicochemical properties. Byun et al. studied the effect of a solvent mixture on the properties of a polylactic acid film. The authors obtained crystallinity, thermal expansion stability, oxygen permeability, and water vapor permeability (WVP) differences by changing solvents between chloroform, methylene chloride, and methylene chloride–acetonitrile [122]. Nevertheless, in the past, PFs intended for wound healing were elaborated principally using organic solvents. However, there is an increased focus on migrating to less-toxic solvents [123] and biodegradable materials [124] to reach a sustainable PFs preparation. In this sense, the use of biodegradable polymers for PFs that are also miscible in water has increased (carboxymethylcellulose, sodium alginate, chitosan, gelatin, xanthan gum, polyvinyl alcohol, polylactic acid, polycaprolactone) [46,84,125].

**Table 3:** Control parameters of PFs.

PF	Properties	Dimensions	Organoleptic Properties	Mechanical Properties	Ref.
-Chitosan 1% (*w*/*v*) -Mansoa hirsuta fraction (MHF) 1.5% (*w*/*v*)	-Advanced healing -Re-epithelization -Cell proliferation -Collagen formation	-Film samples: 5 × 15 cm strips -Thickness: 26.57 ± 2.052 μm	-Smooth and continuous surface	-TS: 22.60 ± 2.79 MPa -EB: 68.75%	[116]
-Soy protein isolate 5% (*w*/*w*) -Glycerol 35% (*w*/*w*) -Glyoxal 1% (*w*/*w*) -Drug 3% (*w*/*w*)	-Analgesic: bupivacaine -Antibiotics: gentamicin, clindamycin	NA	-Soft film (drug incorporation had a softening effect	-YM: 342.5 ± 95.9 MPa -EB: 73.1 ± 35.7% -TS: 17.2 ± 2.8 MPa	[119]
-Chitosan 1% (*w*/*v*) -Carbopol 0.5% (*w*/*v*) -Glycerin 5% (*w*/*v*)	-Antibiotic: mupirocin	-Thickness: 0.504 ± 0.018 mm	-Swelling index: till 900% after 24 h -Moisture loss: 1.120 ± 2.067%	-TS: 0.695 ± 0.11 N/cm^2^ -EB: 211.763 ± 27.119 N	[38]
-Chitosan 1% (*w*/*v*) -Tween 80 0.1% *(v/w)* -H. perforatum oil 0.25–1.5% (*v*/*v*)	-H. perforatum oil: anti-inflammatory, antimicrobial, antioxidant agent, wound healing, and pain relief effect	-Thickness: 0.033–0.066 mm (0.066 ± 0.0029 the one of 1.5% oil *v*/*v*)	-Smooth surface -Transparent and colorless	-TS (without oil): 44.6 Mpa -TS (with 1.5% (*v*/*v*) of oil): 14.8 Mpa -EB (without oil): 7% -EB: 8–21%	[115]
-Chitosan 1% (*w*/*v*) -Bentonite 0.5% (*w*/*v*) Ratio CS:BN: 1:1–6:1	-Bentonite: antimicrobial activity	-Thickness: 17.50 ± 5–42.50 ± 9.75 μm. -WVTR: 1093 ± 20.5–1954 ± 51 g/m^2^/day	-Porosity: increased from 78% to 88% with BN	-Folding strength: 145.25 ± 2.21–289.50 ± 0.57	[117]
-Keratin:Fibrin: Gelatin 1:1:3 ratio (K:F:G)	-Mupirocin: topical antibiotic	NA	-Smooth surface	-EB: 3.61% -TS: 9.48 Mpa	[113]
-Chitosan 1% (*w*/*v*) -PVA 5% (*w*/*v*) -Glycerol 10% (*v*/*v*) -Glyoxal 5% (*v*/*v*)	-For biomedical application	-Thickness: 0.784 mm -Swelling: continuous swelling for 1st and 2nd h (4 h: 1.8 g)	-Smooth surface -Porous, rough surface in a non-crosslinked film	-Hardness value: 53.8 -TS: 0.74 MPa -Maximum load: 0.92 N -EB: 34%	[126]
-Poly(3-HO)/n-BG nanocomposite system -n-BG: 17% for 5 wt% or 9% for 10 wt% polymer solutions	-Matrix support for skin tissue	NA	-Smooth surface	-YM: 3 ± 1–4 ± 1 MPa -TS: 3.3 MPa. -EB: 222 ± 6%–236 ± 10%	[127]
-Sodium alginate (SG): Pectin (PC) solution (5%) (1:1 *w*/*w*) -Glycerol 7% (*v*/*v*) -TP: 5–25 mg/mL	-Tridax Procumbenns (TP): biodegradable, biocompatible, and antibacterial hemostatic agent	-Swelling: up to 251% within 15 min in PBS. -Thickness: 0.193 ± 0.002 mm–0.278 ± 0.002 mm	-Pale yellowish to green-brown color	-TS: 5–6 MPa. -EB: 70% -WVTR: 1500–2000 g/m^2^/24 h	[121]
-Soy protein isolate (SPI) 8.5% (wt) -PVA 10% (wt) -Glycerol 5% (wt)	-Potential wound healing application	-Thickness: 50 μm. -Swelling: 69.243 ± 22.7% -WVTR: 266.7 g/m^2^ day^−1^	-Smooth surface	-TS: 0.85 MPa -EB: 2.0825%	[120]
-Gellan gum (GG): 16 mg/mL -Glycerol: 66 mg/mL -Silibinin nanocapsules: 10 mL	-Silibinin (SB): hepato-protector, antioxidant, and anti-inflammatory	-Thickness: 19 ± 2 μm. -Swelling: 5.11 ± 2.40%	-Transparent -Smooth and continuous surface	-TS: 3.70 ± 0.26 MPa -EB: 4.71 ± 0.37%	[128]

NA: not available, TS: tensile strength, EB: elongation at break, and YM: Young’s modulus.

## 7. Principal Applications of Films for Wound Healing

Films can offer several advantages in different stages of wound closure. During the hemostasis phase, PFs can be a physical barrier to prevent blood loss and provide a scaffold for immune cells, cytokines, and growth factors [15,16,18]. In the inflammation stage, PFs can act as drug carriers, effectively controlling infections and inflammation by targeting the delivery of antibiotics and anti-inflammatories, enhancing the body’s natural defense mechanisms to promote wound healing [21,22]. During the proliferation phase, PFs may help to promote essential processes such as angiogenesis, the generation of granulation tissue, collagen deposition, re-epithelialization, and wound contraction [16,25]. Furthermore, in the remodeling phase, PFs may aid the transition from type III collagen to type I, enhancing wound contraction and the reorganization of collagen fibers [16]. However, further research is required to understand PFs-based therapies for wound healing.

As mentioned, PFs provide a range of benefits, from hemostasis to remodeling phases. Moreover, the main application of PFs varies depending on the treatment of the different wound types. Its versatility is due to the range of polymers available (natural and synthetic) and the ease of industrial scaling for their manufacture [59]. In addition, a wound dressing is appropriate for its antimicrobial properties because infections can compromise skin healing [129]. *E. coli*, *B. subtilis*, and *P. aeruginosa* are the most common microorganisms in wound infections. Therefore, dressings are a barrier to preventing microorganism colonization [130]. For example, when chitosan PFs are blended with cellulose, this can inhibit the growth of *E. coli* and *S. aureus*, enhancing wound repair and epithelial regeneration in wound and burn infections [131,132].

Wound dressings are efficient when carefully considering the causes and consequences of tissue damage. For instance, the leading cause of second-degree burns is the loss of the epidermis. If the wound is too dry, it becomes desiccated, and healing is delayed [133]. Ideal dressings should avoid or remove any inhibitors of wound healing like bacteria, exudate, and trauma [134]. Hence, wound dressings must provide a non-toxic, moist atmosphere to stimulate keratinocytes for faster healing and to provide a custom gaseous exchange [96].

Also, the prevention of wound desiccation results in the formation of an electrical potential between the moist environment of the wounded tissue and the drier area of the non-wounded surrounding tissue. Then, it stimulates the migration of epithelial cells toward the wound site. The expression of growth factors on fibroblast cells also increases after electrical stimuli are provided [44].

Films can be used as a primary or secondary dressing for hydrogels or foams to treat partial-thickness wounds without (or minimal) exudate, necrosis, and infection [34]. As mentioned, films can be used in superficial burns (first and second degree), stage 1 and 2 pressure ulcers, skin grafts, the prevention of skin breakdown, and post-surgical wounds, as they allow for easy wound monitoring [34,135]. Additionally, PFs have a drug delivery application commonly used in pharmaceuticals to penetrate certain drugs into the skin [136]. Specific characteristics needed for the applications of PFs are mentioned in Table 4.

PF possesses advantageous characteristics for wound healing (Table 4). However, due to their limited availability, researchers have noted that wound dressing films must contain various active substances or APIs to produce or increase a specifically intended impact on the wound [36]. These APIs are loaded into PFs, and therapeutic effects on the wound, such as antimicrobial, antioxidant activity, and wound healing acceleration, have been reported [137,138,139]. Some natural compounds are curcumin, epigallocatechin gallate, pomegranate peel extract, and propolis ethanolic extract [36].

Recent studies have evaluated the wound healing properties of curcumin (Cu) in PFs. For example, Cu-grafted hyaluronic-acid-modified pullulan film (Cu-HA-SPu) was promising for the healing process, providing antibacterial and antioxidant properties, and thus indicating that the film is beneficial for cell proliferation and wound healing [139]. Furthermore, other APIs can be loaded into the wound films like antibiotics, including gentamicin, ciprofloxacin, and tetracycline hydrochloride [36,140]. Studies have shown that using gentamicin (GM) in PFs fabricated with hemicellulose, gelatin, and glycerol as a plasticizer exhibited a better antibacterial effect for antibiotic delivery into infected wounds [141].

The use of nanosystems in PFs has been widely studied. Nanosystems incorporated in PFs are used for controlled drug release in the wound environment or tissue. Bardania et al. (2020) [114] synthesized silver nanoparticles (AgNPs) incorporated into PEG/PLA film and showed intrinsic antibacterial and antioxidant activities for wound healing [142]. Another recent study by Kim et al. (2020) [115] described the development of films loaded with various materials. For example, nitric oxide (NO) is a therapeutic agent in CS and S-nitroso glutathione (GSNO) films. The wound healing efficacy of the film was confirmed, revealing re-epithelialization and the reconstruction of the wounded skin, and enhanced antibacterial activity [93].

Furthermore, PFs have also been used in compound wound dressing, combining multiple layers of dressings, to provide adequate healing for a particular type of wound. For example, since films lack absorption as one of their principal properties, they can be combined with foams to counteract this issue [44]. PFs have also been used to make non-contact radiant heat bandages which consist of a heating element, a power source, and two layers of PF. The film protects the wound from bacteria while the heat radiated on the wound promotes dermal blood flow, oxygen tension, and resistance to infection [44].

The applications of PFs are pervasive; hence, films are expected to stand out as novel tools for new-generation wound healing treatments. However, their advantages and parameters to strengthen also depend on the composition of the film and its final purpose. Therefore, the following section will present current commercial medical films for wound healing and some perspectives necessary to enhance research on PFs [33,36,58,59,60].

### 7.1. Current Medical Applications

Over a century of clinical trials evaluating the effectiveness of films in wound healing have been registered in the NIH [143]. Furthermore, several commercial devices, including EktoTherix™, Tegaderm Film^®^, and Ugytex^®^, have demonstrated beneficial results in various applications. For instance, they have effectively treated wounds resulting from the surgical ablation of non-melanoma skin cancers [144], and in promoting anti-infection, anti-oxidative, and conductivity properties to enhance wound healing [145]. Additionally, these films have shown promise in repairing vaginal prolapse [146]. Films in wound healing are crucial as they protect the wounds and facilitate the regeneration of the skin and epidermal tissues. Therefore, it is imperative to continue research efforts to develop innovative products that can aid in wound healing and enhance the overall quality of human life.

### 7.2. Future Perspectives and Areas of Improvement

It is important to note that the studies described in this context primarily have a scientific nature and represent preclinical research involving in vitro and in vivo studies, primarily conducted on rats. For instance, some of these studies evaluate the antibacterial activity of wound dressing materials against *E. coli* and *S. aureus*, and the characterization of antibacterial films loaded with gentamicin [36,44]. These findings are crucial for understanding the potential effectiveness of these materials and formulations. However, additional research, including more clinical trials, is necessary to assess their safety and efficacy in human patients [131,137,138,139].

Given the growing concern over antibiotics in PFs films and antimicrobial resistance, incorporating antimicrobial agents in wound dressings, such as honey or chitosan, presents a promising approach for preventing infections and reducing the reliance on antibiotics [147]. This use of PFs enables the controlled release of antibiotics, enhancing their therapeutic efficacy. However, the overuse and misuse of antibiotics can lead to the development of antibiotic resistance in bacteria. Therefore, further research is necessary in this field to ensure the safety and effectiveness of antibiotics in wound healing films [147,148].

Furthermore, in addition to the considerations regarding antimicrobial stewardship, PFs pose additional challenges, particularly when used as film dressings. Some individuals may find these dressings unappealing due to their transparency, as they may prefer not to have a visual of the wound [33]. Moreover, they are unsuitable for highly exudative injuries because the accumulated fluid can potentially damage newly differentiated keratinocytes, resulting in pain, epidermal damage, and painful removal [149]. Additionally, if the dressing is not sealed correctly, exudate leakage is risky, causing discomfort and necessitating frequent dressing changes [134].

When using film dressings, several important considerations should be taken into account. They are unsuitable for fragile skin, should be avoided in small children, and are not recommended for high exudating or infected wounds. Painful removal is possible, and the application may require prior painful cleaning. Also, these dressings need to be changed a few times a week.

Furthermore, as mentioned, PFs should be regularly evaluated due to their ability to allow oxygen penetration, inhibiting anaerobic bacteria growth. However, their impermeability to liquids can trap bodily fluids in the wound, promoting autolytic debridement but potentially leading to exudate accumulation and making the surrounding tissue prone to maceration [150]. Also, it is important to consider the thickness and material of the film, as they play crucial roles in achieving an adequate mean vapor transmission rate (MVTR), which determines the amount of water vapor escaping through the film. A low MVTR can result in the buildup of exudate [150].

While numerous studies have explored the clinical potential of films and their active ingredients (Table 5), there is still much more to explore regarding their efficacy, biocompatibility, formulation development, and scalability for large-scale applications. Therefore, further research is needed to understand and harness the potential of these wound healing films fully.

## 8. Conclusions

Polymeric films as material for wound healing are among today’s most widely used preparations. Even just the physical cover is already a promoter of wound closure. Polymeric films offer a wide versatility in composition, and, thus, medical applications. The solvent casting method is a process that is easy to implement; however, it includes different critical variables that must be adequately supervised. In detail, it is essential to guarantee a quality product through the prevention of possible interactions between the components in the prefabrication stage, which are mainly due to solubility incompatibility. The participation of the plasticizer is crucial to offer suitable mechanical properties. At the same time, the rheological properties during the solvent process are important for proper molding. The temperature, drying speed, and aging process are elemental in the drying process to ensure suitable physical properties are obtained. Obtaining new polymers or combinations of existing polymers allows the exploration of new manufacturing strategies.

Although the solvent casting method has been widely explored, new challenges persist and appear. The first of these is the close monitoring of the aging stage and the search for new conditions that may allow an improvement in functionality without reformulation. The second is closely monitoring the microscopic architecture, for example, the presence and control of pores, to ensure adequate robustness of the process. Third, although it depends on the type of lesion, bioabsorbable, transparent wound dressings with substances that are not antibiotics and that counteract possible infections are highly desired in clinical practice due to their ease of application and wound monitoring. Finally, there is a continual and more frequent readjustment of the toxicity data for general-purpose and pharmaceutical plasticizers. Some traditionally used plasticizers are highly risky to health; therefore, there is a greater limitation of manufacturing options, and thus the need to discover new plasticizers or suitable combinations of existing ones arises.

## Figures and Tables

**Table 1:** Types of polymers used in polymeric film casting.

Type of Polymer	Examples	Properties	Ref.
Natural	Chitosan, hyaluronic acid, starch, silk fibroin, sericin, keratin, sodium alginate, gelatin, collagen, zein, cellulose, and konjac glucomannan	Biocompatibility, biodegradability, high disponibility, healing properties, permeability, inertness, and bioadhesiveness	[36,37]
Synthetic	Polyvinyl alcohol, polyacrylic acid, polycaprolactone, polyethylene glycol, polyvinylpyrrolidone, polylactic acid, and polydimethylsiloxane.	Resistance, flexibility, structure, high degree of polymerization, thermo-responsiveness, hydrophilicity, and occlusivity	[36]
Blended	Chitosan + pectin	Fluconazole drug administration, antifungal, vaginal film, fast-dissolving	[41]
	Arrow root starch + carboxy methyl cellulose (CMC)	Glipizide in vitro drug administration, buccal film, thin, mucoadhesive, increased tensile strength, clear, biodegradable, edible, smooth	[42]
	Chitosan + PVP	White coloration, reduced mechanical strength (brittle and easier to tear than chitosan film), increased water vapor permeability, reduced antimicrobial properties (>50% PVP)	[40]
	Alginate + Sago Starch + silver nanoparticles	Reduction in inflammation, faster healing, greater tensile strength, porous (good absorption and oxygen exchange)	[43]

**Table 2:** Polymeric films obtained using the solvent casting method.

Polymer	Formulation (% *w*/*v*)	Application	Outcomes	Ref.
Chitosan (Ch)	-4% Ch stock -6% oxidized SA stock -1:2 Ch/SA solution	-Drug delivery	-Non-cytotoxic -No cutaneous reaction after 72 h of in vivo subcutaneous injection -Cytocompatibility in vitro	[63]
Guar gum (GG)	-1% GG -0.5% PVP/0.5 to 5% propylene glycol (PG) as copolymers -200 to 1000 mg Lysine Clonixinate (LC)	-Periodontal treatment	-Highly homogeneous structure -Prolonged API release -Suitable swelling behavior	[64]
Methyl cellulose (MC)	-3% MC -2% SA as copolymer -1 to 5% Montmorillonite (MMT)	-Wound healing and antimicrobial properties	-Delayed thermal drug degradation -Increase in film tensile strength -Bacteriostatic properties	[65]
Sodium alginate (SA)	-4% SA -2% PVA/PVP as copolymers -10% Glycerol/12% PG as plasticizers -Curcumin-loaded PCL nanoparticles	-Wound healing	-High absorbency capacities for exudate removal -Gradual release of the drug -High adherence -Pores with controlled dimensions	[66]
Xanthan gum (XG)	-10% XG -1% Glycerol as a plasticizer -300 mg metronidazole	-Treatment of vaginal infections	-Less frequency of administration compared to conventional treatments. -High adherence -Adequate pH	[67]

**Table 4:** Advantages of film dressings.

Characteristic	Advantages	Ref.
Transparency	Easy assessment of the wound	[33,36,58,59,60]
Impermeability	Effective barrier to water and bacteria	
Porosity	Transmit water vapor from beneath the dressing to the external environment	
Structure	Varying thickness Different sizes and shapes Light Elastic Quickly adherent to injuries	

**Table 5:** Application examples of polymeric films via the solvent casting method for wound healing.

Formulation	Characteristics	Application	Ref.
Collagen 1% + modified microfibrillar carboxymethylcellulose 1% (CMC)	-Good adherence -Flexibility and cohesiveness -Acidic pH and low degree of swelling -Durablility	Swelling and mechanical tests were performed using an artificial wound model (Petri dish and sponge soaked with BSS).	[58]
Polysaccharides extracted from *Hammada scoparia* leaves (PSP) + PVA	-Thickness: 0.0556 mm -In vitro antioxidant activity -Increase the rate of hydroxyproline in the wound site -Accelerate wound closure and re-epithelialization	Tested in a male adult Wistar rat with a circular wound on the dorsal region by excising the skin.	[151]
Chitosan (CS) + konjac glucomannan (KGM) bilayer film	-High biocompatibility -Low cytotoxicity -Transparent -Inhibits growth and penetration of microorganisms -Good thermostability and miscibility -Resist natural deformation of human skin	-Cytotoxicity-viability, and genotoxicity tests were measured using CHO cell line -Proliferation, cytotoxicity-viability, immunofluorescence, and fibroblasts adhesion tests were performed using Human dermal primary fibroblasts	[152]
Chitosan (CS) + hyaluronic acid (HA) at different percentages (1–35%)—2D matrices	-Lower transparency and homogeneity than CS pure -Improved water uptake and surface wettability (HA ≥ 10%) -Hampered water vapor permeability (HA > 5%) -HA affected mechanical properties but provided more flexible matrices (HA = 1–5%) -Fibroblast adhesion and high proliferation (HA = 5%) -Intrinsic antibacterial fouling properties (HA ≥ 5%)	-Proliferation, cytotoxicity-viability, immunofluorescence, and fibroblasts adhesion tests were performed using human dermal primary fibroblasts	[91]
Arabinoxylan (AX) (1.5%, 2%, 2.5%, 3%) and glycerol (2.5%) + sodium alginate (SA) (2%, 2.5%, 3%, 3.5%) loaded with gentamicin sulfate (0.1%)	-Thermally stable -Transparency -Uniform thickness -Smooth surface morphology -Similar tensile strength as human skin -Water transmission rate suitable -Mild water/exudate uptake capacity -Excellent cytocompatibility ->80% GS released in two phases in 24 h (Fickian diffusion mechanism) -Antibacterial effect against *E. coli*, *S. aureus*, and *P. aeruginosa*.	-Cytotoxicity-viability tests were performed using human lung fibroblasts: MRC-5 cells (ATCC CCL-171)	[153]
Polybutyrolactam (PBA) + chitosan (CS) 50%-50% composite film	-Non-toxic -Biodegradable in phosphate buffer saline -Cytocompatibility -Non-allergenic -Higher flexibility -Natural skin-like mechanical properties -Higher water vapor transmission rates -Strength -Promotes cell proliferation	-Cell attachment and proliferation tests were performed using a culture of L929 cells	[39]
Chitosan (CS) + Carbopol + Glycerine loaded with mupirocin	-Bioadhesive -Accelerates the regeneration of the epidermal layer -Improved swelling ability -Promotes epithelialization and angiogenesis	-Ex vivo permeation studies were performed using vertical Franz diffusion cells -Ex vivo bioadhesion and permeation studies were performed using Balb-c mice	[38]
Polyox^®^ (POL) 1% (*w*/*w*) + hydroxypropylmethylcellulose (HPMC), carrageenan (CAR), sodium alginate (SA) or chitosan (CS) (75/25 ratio) and glycerol (GLY) as a plasticizer, loaded with streptomycin (STP) and diclofenac (DLF)	-Homogenous -High swelling -Reduced bacterial infection -Reduced inflammation -Flexible -Transparent	-In vitro, drug dissolution studies were performed using Franz diffusion cells	[154]
Chitosan 2% (*w*/*w*) + PVA 3% (*w*/*w*) + sodium alginate 2% (*w*/*w*) loaded with ornidazole (OD) 1.0 mg/cm^2^	-Excellent light transmittance -Control of water vapor transmission rate -Fluid drainage ability promotion	-Antibacterial studies were performed using *Staphylococcus aureus* and *Escherichia coli*	[155]
Sodium alginate (SA 14.25% *w*/*w*) + PVA 30–25% (*w*/*w*) loaded with vitamin E 3.60% (*w*/*w*) and *Aloe vera* 1% (*w*/*w*)	-High elasticity and strength -Considerable thickness -Biphasic controlled release of vitamin E acetate for more than 12 h -Deep accumulation of vitamin E acetate in the stratum corneum	-In vitro, Vitamin E acetate release studies were performed using modified Franz permeation cells with a synthetic membrane of regenerated cellulose -Tape stripping test with five volunteers (two males and three females)	[156]

## Data Availability

Not applicable.
